# *Adansonia digitata* and *Adansonia gregorii* fruit shells serve as a protection against high temperatures experienced during wildfires

**DOI:** 10.1186/s40529-018-0223-0

**Published:** 2018-02-17

**Authors:** Andreas Kempe, Christoph Neinhuis, Thea Lautenschläger

**Affiliations:** Department of Biology, Faculty of Science, Institute of Botany, Technische Universität Dresden, 01062 Dresden, Germany

**Keywords:** Angola, Australia, Heat-resistance, Fruit shell, Functional morphology, Germination capacity, Seed dormancy

## Abstract

The thick and woody shell of the fruit of *Adansonia* species cannot be explained solely by adaptation to zoochory or hydrochory. Since the trunks of *Adansonia* possess a thick and fire-resistant bark and wildfires occur regularly in its habitat (savannah), we examined with the African *Adanonia digitata* and the Australian *Adansonia gregorii* whether the fruit offers protection against high heat typically experienced in wildfires. Heat-resistance tests were conducted by applying a simple heat test based on known temperature and temperature residence times occurring in savannah fires and complemented by tests to reveal the impact of heat on germination since long-term seed dormancy is known for *Adansonia*. Germination tests with acid treated and heat treated seeds were performed to establish if heat also increased germination rate as effectively as acid treatments have been found to do. Heat was found to increase germination rate, but not as effectively as treatment with acid, therefore fruits exposed to high temperatures experienced in wildfires may have a better chance of germination than fruits that were not exposed to wildfires. The ability of the investigated fruits to protect seeds from high temperatures suggests that wildfires may have played a role in the evolution of the hard-shell structure typically found in *Adansonia*.

## Introduction

*Adansonia digitata*, also known as the baobab, monkey-bread tree or upside-down tree, is widely distributed in and characteristic of sub-Saharan Africa (Wickens and Lowe 2008). The Australian species, *A. gregorii* F. Muell., commonly named boab, occurs in the Northwest Territories of Australia (Wickens and Lowe 2008). The other six *Adansonia* species are endemic to Madagascar (Wickens and Lowe 2008). The noteworthy range of available literature dealing with ecology, traditional and present uses as a medical plant, food and fodder, etc. reflects the extent of attention the genus receives in science.

All *Adansonia* species develop large, ovoid or spherical fruits with a woody pericarp, commonly known as capsules; the botanical term is more precisely amphisarcum: a simple, indehiscent fruit with a pericarp differentiated externally into a dry crust and internally into one or more fleshy layers (Stuppy 2004).

**Fig. 1:**
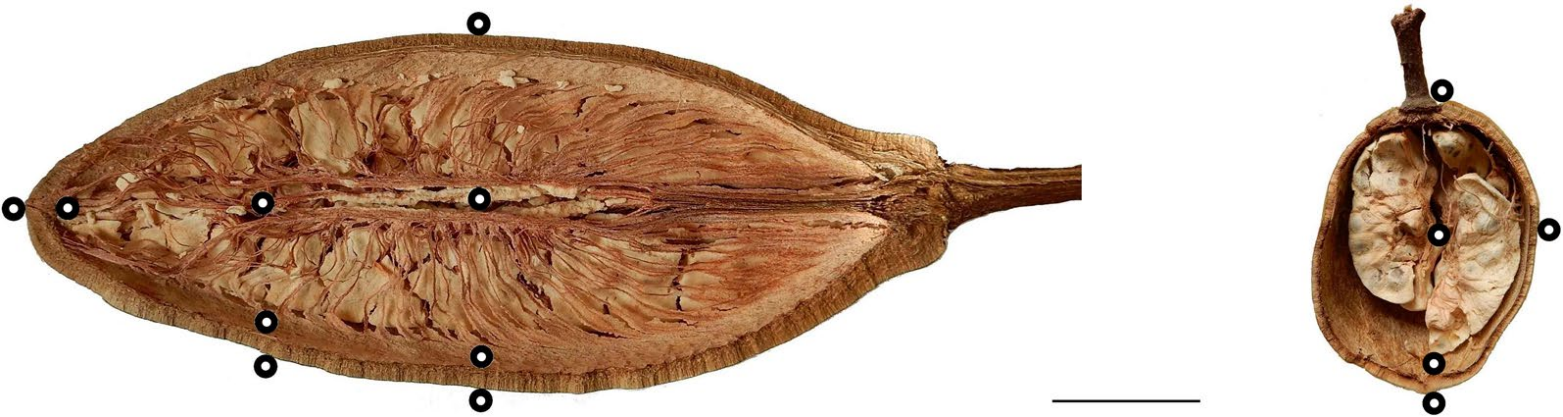
Bisected fruits and position of nine thermocouples in *A. digitata* and five thermocouples in *A. gregorii.* Scale bar 50 mm

The shape of *A. digitata* can vary considerably depending on distribution area (Munthali et al. 2012; Gebauer and Luedeling 2013). In Angola, the ripe fruits are rather elongated, ranging in length between approx. 30 and 65 cm. The hard woody fruit shell has a thickness of approx. 6–10 mm. The fruits of *A. gregorii* are smaller than *A. digitata* and typically reach 15 cm in length and 10 cm in diameter, with a shell thickness of 3–4 mm. Fruits of both species contain a light beige fruit pulp, which appears quite tough, chalky and crumbles when dried (Fig. 1).

In spite of the fact that the baobab is a useful plant with plenty of applications, the shell of *A. digitata* has not yet been the subject of investigations. Our own microscopic investigations and analysis of composition in terms of cellulose, hemicellulose and lignin content is provided in the “Results” section. Detailed anatomical investigations of *A. gregorii* have been conducted by Bleechmore (2002) and were briefly summarised in the “Discussion”.

The study was to investigate if the size and shape of baobab fruit play a role in protecting the seed from the heat experienced in wildfires. Two hypotheses about its woody fruit shell were initially considered: zoochory and hydrochory as two modes of seed dispersal. A hard or thick fruit shell can restrict access to the fruit pulp and hence the seeds to a particular distributor. Further, a woody fruit is able to float and drift to new shores. In order to explain the fruit’s form and function in terms beyond zoochory and hydrochory, we also investigated whether the shell serves as heat protection during wildfires for seeds, bearing in mind that in savannahs, species must cope with wildfires. Moreover, heat may even enhance seed germination.

### Zoochory and hydrochory

Wickens and Lowe (2008) noted that elephants, baboons, ungulates, bush pigs, small mammals, birds and other animals all eat the fruit pulp of *A. digitata*, either when capsules fall and break apart or sometimes when the shell has already opened on the tree, when the content is accessible to bats and birds. In Angola, rural people have reported that rat-like animals, most likely rock rats, gnaw at fruits hanging on the tree. Marsupials such as kangaroos and wallabies are dispersers in Australia (Wickens and Lowe 2008). Kamatou et al. (2011) noted that seed dormancy is broken when the seeds pass through the digestive tract of animals that consume the pulp. The seed shell is probably partly digested and thinned, which helps the embryo to break through. Similarly, Esenowo (1991) found that the most effective method to break dormancy was scarification of seeds.

It has been reported that Malagasy baobab fruits may be dispersed by marine hydrochory (Cornu et al. 2014). These authors were able to show that fruits found on the beach could spend a long time in the sea with little effect on their germination potential. Local dispersal by floodwater of rivers has been described for both *A. digitata* and *A. gregorii* (Wickens and Lowe 2008). Tsy et al. (2009) showed successful germination of *A. digitata* after storing seeds in sea water for 6 months (> 97% germination rates for each of four fruits immersed in seawater and maintained in dry conditions). Our own experiments with *A. digitata* in which five fruits were stored in a tub of salty water (3.5% salt content) and maintained a shaking plate to simulate wave action revealed that three fruits decayed after approx. 4–7 weeks (unpublished data). Once the fruit shell cracks, seeds were released and sank. Also, seed shells and seeds of the two fruits that remained floating decayed inside the fruits. Hence, long distance marine dispersal in *A. digitata* remains to be verified. Nevertheless, Baum et al. (1998, 2004) and Tsy et al. (2009) concluded long distance marine dispersal on the basis of phylogeography, at least for the common ancestor of present baobab species.

### Distribution and fire adaptation

The distributions of *A. digitata* in Africa and *A. gregorii* in Australia coincide with the tropical savannah vegetation zone (Beard 1967; Owen 1974; Walker and Gillison 1982; Wilson 1988; Fukarek et al. 1995; Wickens and Lowe 2008). At the same time, it is known that fire plays an important ecological role in savannahs (Trollope 1984; Schüle 1990; Pausas 2015). Trees from fire-prone habitats therefore possess well-adapted barks to protect the cambium and to withstand wildfires. The key features are bark thickness and bark water content (Pinard and Huffman 1997; Chapotin et al. 2006; Lawes et al. 2011a, b; Rosell et al. 2014). *A. digitata* is recognized as well-adapted to fire, having a thick and fire-resistant bark (Owen 1974; Sidibé and Williams 2002; Chapotin et al. 2006; Watson 2007). Regeneration capacity after fire has also been described for *A. digitata* by e.g., Owen (1974) and Patrut et al. (2010). Some authors have noted that juvenile plants prefer fire protected sites (Duvall 2007; Venter and Witkowski 2013), whereas other authors assessed the lethal threat of fire to baobabs as limited because of lack of fuel load, notably in Zimbabwe (Romero et al. 2001) and South Africa (Venter and Witkowski 2013). Especially when trees are older than approx. 15 years, trunks are large enough to withstand fire and thus mortality due to fire decreases drastically (Johansson 1999). Juvenile trees of both species are capable to resprout from tuberous roots after fire (Bowman 1997; Nano and Clarke 2011; own observations).

### Wildfire characteristics

The severity of wildfires depends on several parameters: fuel load, fuel distribution, compaction, moisture, air temperature, relative atmospheric humidity, wind etc. Early or late dry season fires differ significantly due to fuel load and drought (Trollope 1984; Williams et al. 1998). Notably, fire intensity is strongly correlated to fire frequency: frequency reduces intensity (Schüle 1990). It is known that surface fires are most common in savannah, while crown fires occur only under extreme fire conditions (Trollope 1984). Savadogo et al. (2007) and Dayamba et al. (2010) documented fire temperatures in relation to height for savannah woodlands in Burkina Faso. Regardless of season, maximum temperatures appeared near ground level, whereas at 1.5 m temperatures decrease to approx. 175 °C and drop below 100 °C near 5 m (Savadogo et al. 2007; Dayamba et al. 2010). In the same studies, temperature residence time above 60 °C was less than 2 min (late season) and less than 1 min (early season) at 1.5 m. In comparison, Miranda et al. (1993) measured up to 260 °C with annual fire frequency but as much as 650 °C at 1.6 m in Brazilian Cerrado fires when fire had been absent for 15 years; air temperature decreased to approx. 100 °C within 4 min. These information provided the basis for our own heat tests.

### Approach of a heat test

We developed heat tests and measured temperatures over time inside and outside of fruits. We based our temperatures and heating duration on the data cited above, bearing in mind that a broad range of these two parameters can occur. In general, any heat applied to the outside of an object will be transmitted to the inside after a specific period of time that depends on the insulating properties of the material. We hoped to determine how long the fruit shell would insulate the inner tissue from a given heat load, given that bush fires occur over a period of just a few minutes (Vines 1968; Miranda et al. 1993; Savadogo et al. 2007). If the fruit shell is capable of delaying heat transmission, seeds can survive a bush fire undamaged if not exposed to naked flames.

We wished to simulate the conditions of a real savannah fire for fruits hanging on a tree, since safety and financial factors precluded a large-scale fire. Thus, for our heat test we abstracted the heat source and used charcoal on a grill that delivered a constant and regular heat and which could be repeated. Hence, heat was supplied primarily as radiation, whereas flames and convection were neglected. Consequently, the results serve primarily as the basis for discussion.

### Germination and impact of heat

To complete our study, germination rate (GR) was determined to evaluate the impact of the heat on seeds. In general, temperatures above 60 °C are considered lethal for living tissues (e.g., Hare 1965; Vines 1968), although frequently that does not apply to seeds. For example, Esenowo (1991), Auld and O’Connel (1991), Gashaw and Michelsen (2002), and Dayamba et al. (2008, 2010) all reported that seeds from African and Australian savannah plants can withstand temperatures ranging from 60 to 200 °C, for at least 1 min. Esenowo (1991) argued that heat seems to play an important role in germination of *A. digitata*. He demonstrated that seeds achieved higher GR after 40 min soaking in 60–70 °C tempered water than soaking in cold water or after 1–5 min soaking in 80 °C tempered water, respectively. In contrast, Johansson (1999) documented that GR of *A. digitata* were no higher after treatment in boiling water compared to soaking in cold water and reported no germination after 5 min dry heat treatment at 100 °C. Maghembe et al. (1994) found GR for *A. digitata* greater than 80% regardless of whether seeds were untreated or treated with cold or hot water. Turner and Dixon (2009) documented GR for *A. gregorii* below 25% after treatment with boiling water for various durations. In general, fire is believed to help break the dormancy of *Adansonia* seeds (Watson 2007).

In order to break dormancy, experiments simulating passage through an animal’s digestive tract have been carried out by soaking seeds in acidic solutions; the results have shown positive GR for both *A. digitata* [60–90% GR using the acids HCl, HNO_3_, H_2_SO_4_ and various durations of soaking (Esenowo 1991), 2% GR after soaking in HCl (Johansson 1999), 70–100% GR after various durations of soaking in concentrated H_2_SO_4_ (Razanameharizaka et al. 2006), 5–97% GR after various durations of soaking in concentrated H_2_SO_4_ (Niang et al. 2015)] and *A. gregorii* [85–100% GR after various durations of soaking in concentrated H_2_SO_4_ (Turner and Dixon 2009)].

## Methods

### Material

Six fruits of *A. digitata* (two fruits per individual) were collected in the province Uíge in northern Angola (S8°33′05.9″, E13°45′07.8″, 139 m) in March 2015, at the end of the rainy season. The fruits were between 34 and 52 cm long with a diameter of 11.4 up to 14.8 cm at the widest part. They had a volume between 2.1 and 6.0 dm^3^ and a dry weight between 0.7 and 1.4 kg. The shell’s thickness ranged around 7.6 (± 1.6) mm.

Northern Territory Herbarium (DNA) in Palmerston, NT, Australia provided 20 ripe *A. gregorii* fruits in May 2014. The fruits were collected along Parry Creek Lagoon Road and Flametree Nursery, Riverfarm Road Kununurra from two trees, 9 and 10 m high with trunks of more than 1 m diameter at breast height. Six average fruits were used which had a length between 9.1 and 15.0 cm with a diameter of 7.2 up to 8.1 cm at the thickest part. The volume ranged between 0.23 and 0.42 dm^3^ and the dry weight between 62 and 142 g. The shell’s thickness was 3.0 (± 0.4) mm.

### Anatomy

Fruit size and shell thickness were measured with yardstick with an accuracy of 1 mm and calliper with an accuracy of 0.1 mm, fruit volume by water-displacement with an accuracy of 10 ml. Sections of the pericarp were cut by a microtome, stained with Astra-blue/Safranin (Morphisto), and analysed using a light microscope (Carl Zeiss Axioskop 2) and a reflected light microscope (Olympus SZX16). Cell size and cell wall thickness were assessed. The density of shell was determined with five different samples according to DIN EN ISO 1183-1 using a precision balance (Kern, EMB 100-3) and a pycnometer (50 ml, Boro 3.3, Brand) with an accuracy of 0.01 g and 0.05 ml, respectively. Cellulose, hemicellulose and lignin content were determined by the Institute of Plant and Wood Chemistry (TU Dresden) according to standardized methods by Klason (1920), Kuerschner and Hoffer (1931), and Poljak (1948). Bremer et al. (2013) provide details of the extraction process.

### Heat test

**Fig. 2:**
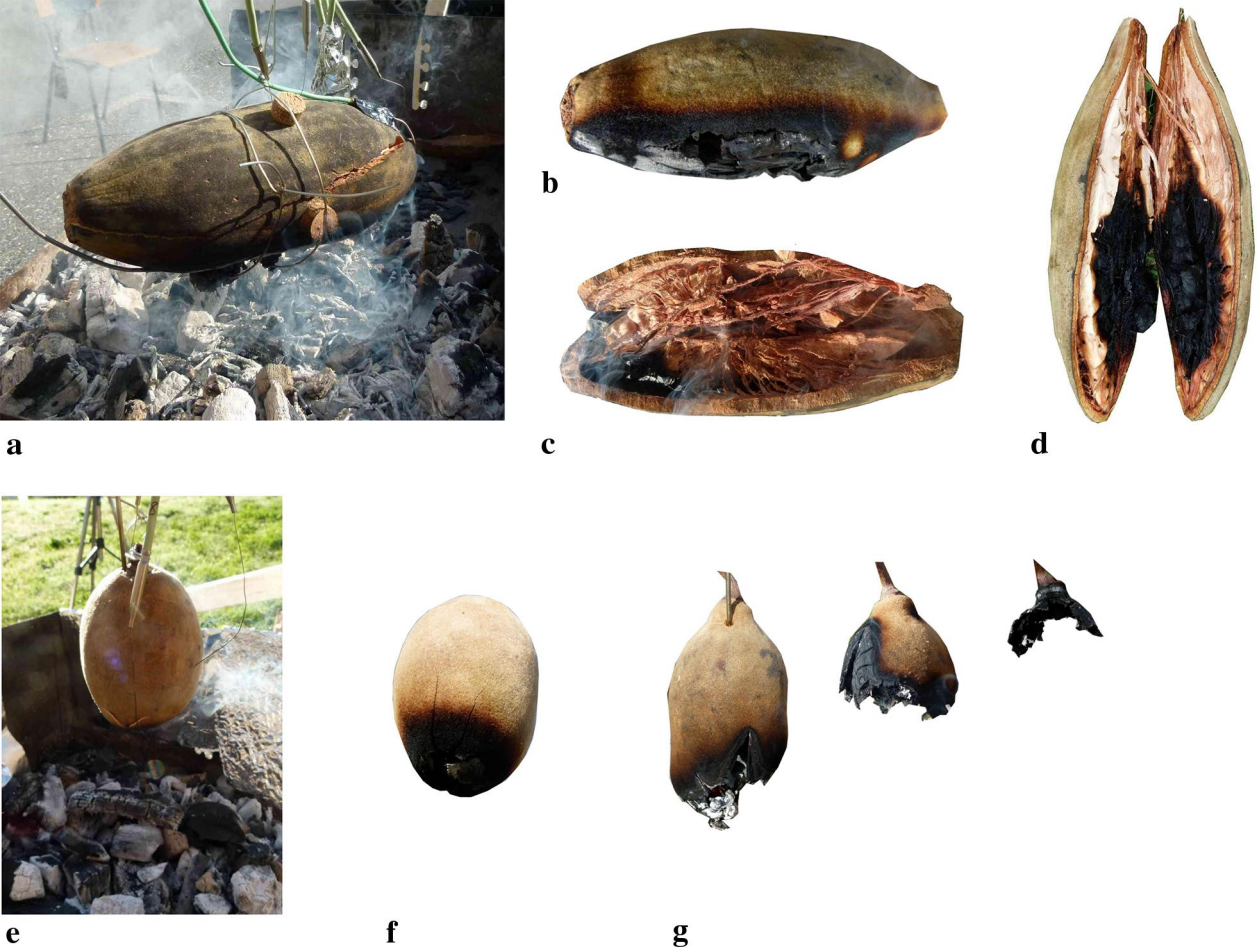
**a** *A. digitata* above glow with cracked shell by heat. **b** Decomposed fire-facing side after test. **c** Same fruit, view inside after test less than 10 min above glow with partly unburned and browned pulp. **d** Another fruit opened with heavily smouldered parts after test more than 10 min above glow. **e** *A. gregorii* above glow. **f** Same fruit, fire facing side after test. **g** Another fruit, in one case, ongoing smouldering of fruit shell (heat source was already removed) with successive shell decomposition and complete loss of seeds. All scale bars 50 mm

To investigate the delay of an experienced outside temperature to the inside of the fruit, temperatures outside and inside the fruit were recorded via thermocouples (tc’s) at several positions (Fig. 1) during heating. Therefore small holes with a diameter of 3 mm were drilled from the top into the shell to insert tc’s. The depth of the holes were measured with a calliper in order to place the tc’s at the right position. Inner tc’s were placed directly at the inner side of the fruit shell and in the mid axis of the fruit. Additional tc’s were placed outside and fixed with tape (Fig. 2). Thermocouples type K (Jumo) according to DIN43710/IEC584 with a range of – 200–1150 °C were used. Temperature and time were logged every second by NI 9213 thermocouple input module (NI USB-9162 USB Single Module Carrier and Software SignalExpress 2012, National Instruments). Temperature gradients and corresponding time were analysed using the spreadsheet of Apache OpenOffice. The fruits were attached with a steel wire (0.5 mm diameter) at a wooden frame over the heat source. All frame elements had a distance of more than 1 m to the heat source. The wires of tc’s were fixed upwards to the frame and then led towards the instruments. Beech charcoal on a grill served as heat source and delivered constant heat for more than 3 h. Heating value is given with 25–35 MJ kg^−1^. The amount of used charcoal determines the temperature during burning. We used always the same amount of approx. 4.0 kg which delivered 500–600 °C. Heat transfer by radiation depends on temperature and radiating area. The radiation area was approx. 0.25 m^2^. Thus the rate of heat flow, calculated by Stefan–Boltzmann law, was at least 4–6.5 kW. Between 100 and 200 °C were measured at approx. 10–20 cm above glow, which was the distance between glow and the fruits.

Fruits of *A. gregorii* were placed hanging vertically above heat source, as fruits hang naturally, whereas *A. digitata* fruits were placed horizontally for optimising heat transfer between heat source and fruit. Otherwise significant heating would only have occurred at the tip due to small radiating area and less heat would have reached the top of the fruit. Gradient of temperature over time was recorded during heat exposure and during cooling down as well.

### Germination test

Seeds of three and four heat tested fruits of *A. digitata* and *A. gregorii* were used for germination tests. *A. digitata* seeds were divided into two batches, one from the glow facing side or lower half and one from the upper half. Heat tested seeds were compared to following control sets: soaking of seeds in concentrated H_2_SO_4_ for 10 min with subsequent rinsing in running water for 3 h (Esenowo 1991), in 70 °C hot water for 40 min (Esenowo 1991), cold water for 3 days (Johansson 1999), and no treatment. Therefore further two fruits of *A. digitata* and three fruits of *A. gregorii* were used. 50 seeds of each experimental variant were extracted out of its fruit pulp by cutting with a knife. Thus, 10 variants with in total 500 seeds are used for analysis of *A. digitata* (Table 1) and eight variants and 400 seeds for *A. gregorii* (Table 2).

**Table 1:** Germination rates (GR) of *A. digitata*

Heat treatment		GR (%) (upper, lower side)
Average of T_max_ of inner tc’s (°C) (SD)	Heat exposure time (min:s)	
76 (25)	06:25	3 (0, 6)
198 (103)	12:00	15 (24, 6)
445 (173)^a^	10:00	25 (34, 16)
Control		
No treatment		0
15 min H_2_SO_4_		76
40 min hot water, 70 °C		16
3 days cold water		2

^a^Local smouldering in fruit 3 led to extreme divergent temperatures (maximum of 595 °C at central tc)

**Table 2:** Germination rates (GR) of *A. gregorii*

Heat treatment		GR (%)
Average of T_max_ of inner tc’s (°C) (SD)	Heat exposure time (min:s)	
120 (70)	10:45	2
128 (60)	11:45	14
152 (125)	11:45	10
429 (116)	12:00	0
Control		
No treatment		10
15 min H_2_SO_4_		24
40 min hot water, 70 °C		6
3 days cold water		4

Cultivation took place in the Botanical Garden of the TU Dresden from the beginning of March to the end of September 2016, with greenhouse conditions of an average temperature of 21 °C and an average humidity of 82%. Seeds were irrigated every day. Germination was recorded every week.

Number of germinated seeds were analysed using the spreadsheet of Apache OpenOffice. Significant differences between treatments were analysed by Fisher’s exact test (McDonald 2014).

## Results

### Anatomy—*Adansonia digitata*

**Fig. 3:**
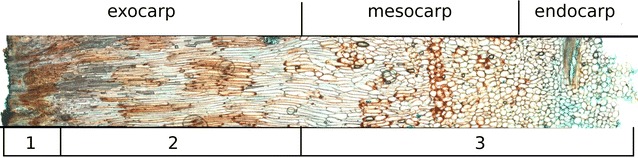
Micrograph of *A. digitata* pericarp; shell thickness is approx. 7 mm

Microscopic investigations revealed three layers of shell composition, which can be classified as exo-, meso- and endocarp (Fig. 3). Different cell dimensions characterized these layers. The outermost exocarp had densely packed, globular, and thick-walled cells, followed by radially elongated and thick-walled cells. Meso- and endocarp consisted of undirected elongated and spherical cells. The inner layer had the smallest and most thin-walled cells.

Density of fruit shell and of pulp was 0.61 (± 0.09), and 0.16 (± 0.01) g cm^−3^, respectively. Due to visible inhomogeneity of *A. digitata*’s fruit shell, lignin, cellulose and hemicellulose were determined for three layers, which, however, did not represent exo-, meso- and endocarp but combined meso- and endocarp and divided exocarp in the outermost and the inner exocarp (Fig. 3). The outermost layer of exocarp consisted of 45% lignin, 21% cellulose and 28% hemicellulose. The subjacent layers counted for approx. 30% lignin, 36–38% cellulose and 33–37% hemicellulose.

### Anatomy—*Adansonia gregorii*

Density of fruit shell and of pulp was 0.55 (± 0.06), and 0.20 (± 0.03) g cm^−3^ respectively. Further anatomy of *A. gregorii* has been investigated by Bleechmore (2002) and is given in the “Discussion” section.

### Heat test

In the first 3 min the temperature gradients were nearly identical for all tested fruits. Once the fruit was placed over the heat source, all tc’s recorded increasing temperatures. The outer temperature on the glow facing side jumped up immediately within seconds to 100–150 °C. The inner temperatures increased slowly and continuously: direct behind the shell faster than in the mid axis.

After 3 min: During exposure to glow the outer lower tc increased further up to 300 °C, whereas the temperature directly behind the glow facing shell eventually remained below 100 °C except in one case up to 110 °C. The temperature of central tc’s did not exceed 50 °C.

6–14 min: All fruits became charred on the glow facing side. The tc’s recorded a local increase up to a maximum of 587 and 240 °C directly behind the shell for *A. digitata* and *A. gregorii* respectively. When approx. 90 °C were attained in the centre of the fruit it was removed from the heat source. One fruit of each species was already removed after reaching 60 °C in the centre. Two *A. digitata* fruits showed cracks of a maximum of 0.5 cm width and 25 cm length. The difference of outside and central temperature was between 188 and 366 °C for *A. digitata* and between 116 and 406 °C for *A. gregorii* at the end of heat exposure.

**Fig. 4:**
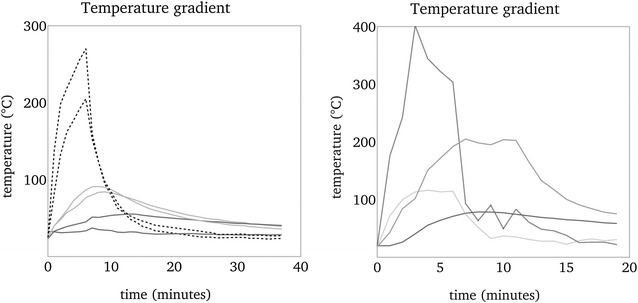
Example of temperature gradients of *A. digitata*, left, and *A. gregorii*, right. Curves are smoothed, average temperature per 1 min. Left: Temperature gradient of 6 tc’s. Each 2 black dotted, light grey and dark grey lines are temperatures outside downwards, inside the shell downwards and in the centre. Heat exposure 06:25. Record time 37 min. Right: temperature gradient of 4 tc’s. Black dotted, light grey and dark grey lines are temperatures outside downwards, inside the shell downwards and in the centre. Grey dotted line is outside sidewards. Heat exposure 6 min. Record time 17 min

End: The heat source was removed. Temperature of outer tc’s dropped immediately nearly to ambient temperature, whereas the temperature inside decreased slowly. Fruit shell and inner parts of the two cracked *A. digitata* fruits started to glow and smoulder within 5 min after removal from heat source. A hole appeared in the shell from which some smouldering seeds fell down. At the same time central temperature increased locally further up to 595 °C after approx. 15 min. At that time recording was terminated. However, half of the fruit remained more or less unburned. In contrast, fruit shell and inner parts of one *A. gregorii* fruit started to glow and smoulder after removal from heat source. This fruit shell smouldered off completely and degraded to ashes (Fig. 2). All seeds from this fruit were lost. Representative gradients are displayed in Fig. 4.

### Correlation of shell thickness, fruit volume, heat exposure time and temperature

**Fig. 5:**
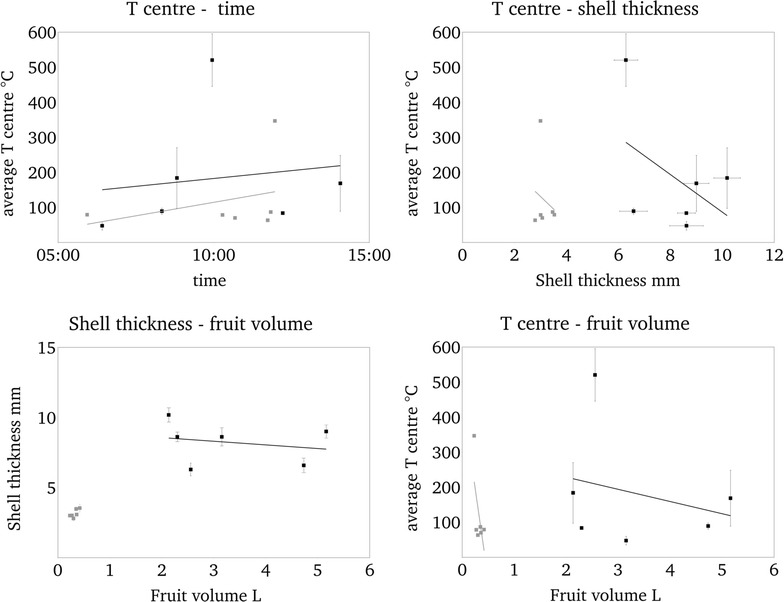
Correlation of temperature in the centre of fruits, fruit volume, heat exposure time, and shell thickness. Black *A. digitata* and grey *A. gregorii*

Heating of the fruit was influenced by fruit volume, shell thickness, and most relevant by heat exposure time (Fig. 5). A long heat exposure caused generally higher temperatures at tc’s (Fig. 5). Importantly, *A. digitata* fruits with the thinnest shells reached highest measured inner temperatures (Fig. 5). Shell thickness and volume were not correlated (Fig. 5). Summing up, the maximum temperature outside the shell ranged between 210 and 490 °C within approx. 6 and 14 min for both species. The most important point was that in three of six *A. digitata* fruits the average central temperature remained below 90 °C within the test time and additionally as well in two further fruits at least at one of both central tc’s. The *A. digitata* fruit with the highest central temperature had the thinnest shell and small volume. Shell thickness of *A. gregorii* was more homogeneous than in *A. digitata* and showed no relation to inner temperatures (Fig. 5). They depended rather on fruit volume. Smallest fruit had highest end temperatures (Fig. 5).

### Germination test—*Adansonia digitata*

Treatment with sulphuric acid had the highest GR (Table 1). Seeds from heat tested fruits tended to germinate more when experienced temperatures above 100 °C. Hot water treatment had as well a positive effect on GR. Temperatures below 70 °C had little impact on GR (Table 1). There was a distinct difference between GR from glow facing side and upper side. Both fruits with higher experienced temperatures exhibited higher GR on upper side. The fruit with less heat exposure showed higher GR on the lower side (Table 1). A percentage of 90% of all germinated seeds sprouted within 6 weeks after sowing.

**Fig. 6:**
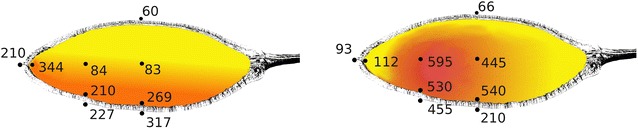
Possible temperature distributions in *A. digitata* approximated from maximum temperatures (°C) at the end of heat exposure. See also charred region in fruit in Fig. 2d. Left: continual gradient. Right: smouldering seat. Yellow: low temperature; red: high temperature

We have to emphasise that given temperatures were only measured very selective with five tc’s inside the fruit. We observed maximum differences of more than 200 °C between two central tc’s during heating, situated 10 cm away from each other. Thus, it was unclear how locally limited a measured maximum temperatures was. We assumed carefully a possible temperature distribution from maximum temperatures at the end of heat exposure of two fruits (Fig. 6). The values of fruit 3 must not mislead the reader that the higher a temperature the higher was the GR. Logically seeds cannot survive these high temperatures.

### Germination test—*Adansonia gregorii*

Except from treatment with sulphuric acid, the GR’s were up to 14% (Table 2). An amount of 75% of all germinated seeds did not sprout until the 12th week, but the earliest germination was observed for acid treatment after 4 weeks. The difference between highest GR after heat treatment (14%) and no treatment (10%) was not significant (P value = 0.48).

## Discussion

In a preliminary work we asked if the shell of *A. digitata* would offer sufficient mechanical properties to restrict access by a frugivore. In particular we analysed two properties characterising rigidity and strength of the *A. digitata* shell employing mechanical stress tests. Young’s modulus was determined by three-point bending test to be approx. 1.0 GPa and Charpy impact strength was measured to be 2.9 kJ m^−2^. Both values were below one tenth of average angiosperm wood (Kretschmann 2010). Young’s modulus for *A. gregorii* fruit is in the same range (approx. 1.5 GPa; Bleechmore 2002). Hence, we concluded that the fruit shell does not serve exclusively as seed protection against animals. In this study we investigated the fruit shell’s capability of protecting seeds against high temperatures during wildfires.

### Heat protection

When we talk about heat protection, we intended to evaluate heat protection for relevant conditions in savannah fires given by temperature-residence time and temperature. Wildfires in savannahs use up its fuel quickly and therefore usually last less than 10 min (Miranda et al. 1993; Savadogo et al. 2007; Dayamba et al. 2010). Heat impact during savannah fires were given with air temperatures ranging between 100 and 260 °C (rarely up to 650 °C) at 1.5 m height and still with approx. 100 °C at 5 m height (Miranda et al. 1993; Savadogo et al. 2007; Dayamba et al. 2010). Usually the lowest fruits of both investigated *Adansonia* species hang not below 1.5 m height. Maximum heights of mature trees of *A. digitata* and *A. gregorii* are up to 20 and 10 m respectively. Since fruits are randomly distributed over the entire tree crown, fruits are in threat of fire at least to a height of 5 m.

Based on that we arranged a fire test using beech charcoal, which had a temperature of 500–600 °C and a radiation area of approx. 0.25 m^2^. Fruits were placed 10–15 cm above the glow. Both *A. digitata* and *A. gregorii* withstood at least 5 and 4 min, respectively, without reaching 60 °C in the centre while surface temperature of the fruits further increased to approx. 300 °C. A temperature of 90 °C in the centre was reached at the earliest after approx. 7 min in one *A. digitata* fruit and later than 9 min in four of six *A. digitata* fruits. This temperature was not exceeded in *A. gregorii* within 10 min. The lethal temperature for seeds is known to be higher than 90 °C (Esenowo 1991; Maghembe et al. 1994; Johansson 1999; Turner and Dixon 2009). The delay of temperature increase inside the fruits clearly demonstrates the shell’s capability to protect seeds against heat. Regarding the fact that savannah fire duration is less than 10 min (Vines 1968; Miranda et al. 1993; Savadogo et al. 2007) the fruit shell delivers sufficient protection for the largest amount of fruits on a tree. Only in case of higher temperatures, close fire distance, and long heat exposure time, fruits may be damaged partially or totally leading to loss of seeds. Concerning our experimental set-up, decomposition conditions for fruit shells could be reached by more than 8 min heat exposure with 10 cm fruit—glow distance and heat source temperatures above 500 °C. When distance to glow were below 10 cm then shells of course decomposed faster.

Strong temperature gradients could be measured inside *A. digitata* fruits during heating. In one case a maximum difference of more than 200 °C occurred between two central tc’s for at least 1 min in case of inner smouldering. Thus, the spongy fruit pulp definitely contributed to thermic insulation of seeds, too. In the case of inner smouldering peak temperatures of almost 600 °C occurred, the smouldering fire did not propagate into the whole fruit. It was striking that approximately the half of the fruit pulp remained unburned (Fig. 2d). Thus, in this case a considerable number of seeds would probably have survived.

We observed furthermore that sometimes shells were already split open on the tree or cracked during fire. Thereupon seeds could easily fall down into the surface fire, where they most probably were destroyed. Wickens and Lowe (2008) described frequent shell cracking for *A. gregorii* and argued that with enhanced seed dispersal (Wickens and Lowe 2008). If this has evolved actually as adaptation for Australian fauna, in our opinion, fire threat should have been limited.

The main differences between both *Adansonia* species investigated here were fruit size and pericarp thickness. The Angolan *A. digitata* fruit volume were approx. up to ten times larger than that of *A. gregorii* and its shell more than twice of the pericarp thickness of *A. gregorii.* But, *A. digitata* exhibits high fruit phenotype diversity in its African distribution range, and size and pericarp can also be similar, even identical to fruits of *A. gregorii*, examined for Sudanese and Malawian baobab populations (Munthali et al. 2012; Gebauer and Luedeling 2013). It is speculative if fruit size diversity has evolved depending on variations of fire intensity. We could not find any data about differing fire intensities and a correlation to fruit size in African *Adansonia*. Furthermore fire intensities in African or Australian savannah does not differ generally (Williams et al. 1998; Vigilante et al. 2004; Govender et al. 2006). Thus differences in fruit size between *A. digitata* and *A. gregorii* cannot be explained clearly by fire threat.

The expected time span until the temperature of 90 °C was reached in the centre of fruits should be shorter for small fruits than for large ones. In our experiments *A. digitata* exposed a greater surface to the glow, which led in consequence to its faster heating up as compared to *A. gregorii*. This was clearly a result of our experimental set-up.

Regarding heat-resistance of the baobab fruit shell, a comparison to fire-resistance of bark in savannah trees is obvious. The main parameter determining a tree’s fire-resistance seems to be bark thickness (Hoffmann et al. 2003; Lawes et al. 2011b, 2013; Rosell et al. 2014; Schafer et al. 2015). Regarding studies on correlations of bark thickness, bark density and fire-resistance of trees, low bark density is not necessarily an indication for fire-resistance (Brando et al. 2012). High bark density due to high bark moisture content may be a good fire protection, as well (Lawes et al. 2011b). Conversely, Bauer et al. (2010) demonstrated that fire-resistance of the bark of seven tree species increased with decreasing bark density. They have argued that a less dense bark type with internal air spaces could be advantageous for fire-resistance by additional insulation effects due to the internal air spaces. Therefore the low density of fruit shell and pulp provide at least a good heat insulation of the seeds.

### Anatomy

Our findings coincide evidently with Bleechmoore’s (2002) results. Shells of both species consist of a very similar three-layered structure, characterized by the different tissue type and cell orientation: “…the exocarp, which is a thick outer wall, comprising radial elongated thick-walled cells; the mesocarp, consisting of thin walled parenchyma cells; and the endocarp, which comprises sclerenchyma cells of both fibres and sclereids.” “The mesocarp comprises the bulk of the wall…, with slightly radially orientated cells until close to the endocarp, where the cell lumen increases and the cells become more randomly orientated.” (Bleechmore 2002).

The mechanism of heat-resistance can be found in the chemical composition of the shell. Even though the shell material resembles wood, it is different from wood with regard to lignin and cellulose proportion. Reviewing 29 papers concerning ignition temperatures of wood, Babrauskas (2001) reported that softwoods, compared to hardwoods, have a smaller content of hemicellulose and a higher content of lignin [up to 34% (White 1987)], resulting in higher ignition temperatures. Hemicelluloses and lignin components are pyrolyzed in the ranges 200–300 °C and 225–450 °C, respectively (White and Dietenberger 2001). The higher lignin content in the outermost exocarp layer *of A. digitata* fruits (up to 45%) helps explain why the fruit may have the ability to protect against inflammability. This is substantiated by the occurrence of highly lignified cells in the exocarp of *A. gregorii*, as well (Bleechmore 2002).

### Germination

Published literature reports differing results about successful germination of *A. digitata* after acid or heat treatment (Esenowo 1991; Maghembe et al. 1994; Johansson 1999; Razanameharizaka et al. 2006; Niang et al. 2015) but without unequivocal results, which treatment could explain seed distribution mechanisms. For *A. gregorii* Turner and Dixon (2009) showed higher germination rates (GR) for acid as well as for heat treatment. It is noteworthy that seeds of both species can withstand immersion in boiling water for up to 3 min without total loss of germination capability (Razanameharizaka et al. 2006; Turner and Dixon 2009) indicating an appropriate adaptation to wildfires.

Our data support a positive effect of heat on GR only for *A. digitata* reaching 25% while seeds without treatment did not germinate. Sulphuric acid treatment, however, resulted in 76% GR and 24% for *A. gregorii*, whereas heat treatment or no treatment did not differ significantly in the latter, GR was only up to 14 and 10% respectively.

Since acid treatment is assumed to simulate animal’s digestion, our results substantiate the hypothesis of animals as seed propagators of *Adansonia*. Using only 2–5 tc’s for inner temperature measurement provide only rough information about min./max. temperature distribution and gradients within fruit pulp. We can only carefully estimate from one fruit of *A. digitata* that heat treatment below 60 °C had no positive impact on germination. An upper temperature limit was not detectable due to a smouldering fire pocket close to the tc location in one case.

The seed shell is hard and up to 0.6 mm thick. Acid treatment thins the seed shell, facilitates water entering the seed, and helps the embryo to break through. Heat treatment may also crack and fissure the seed shell and contribute to an easier breaking through of the embryo as well but not as effective as acid. However, improved germination due to heat exposure does not contradict the argument of heat protecting fruit shell and can just be a side effect.

In our study the influence of the seed shell to thermic insulation was not investigated. It can be assumed that the seed shell contributes to heat protection. Which temperature eventually occurs in the inner of a seed is not known. Therefore lethal conditions for the seeds could not be derived from our experiments.

Determining characteristic values of flammability or thermal conductivity of standardised fruit shell samples were probably more meaningful, but we wanted to evaluate germinating capacity after realistic exposure to heat concerning the entire fruit. This led to the disadvantage that six fruits each of both *Adansonia* species did not deliver enough data to draw additional conclusions. This would require fruits of the same size, volume or shell thickness.

Furthermore using charcoal on a grill as heat source did not reflect a realistic savannah fire. Glow delivered radiation but only a small contribution of convection. Flames have large turbulent convection effects, influencing the speed at which the object is heated. Key sizes of radiation are area and temperature of heat source, i.e., a larger heat source area results in faster heating and higher temperatures. Forced convection appears due to up-current and wind. Consequently faster air movement causes greater heat exchange. Thus, our methodological approach clearly affects the heating time. As a result, the considered heat exposure times may be overestimated by a factor of two. On the other hand, the horizontal positioning of *A. digitata* fruits did not correspond to the natural exposure to fire. In fact, exposing the lateral surface to the heat source resulted in faster heating up of the fruit. Thus, our heat exposure times may be underestimated for *A. digitata*.

## Conclusion

We investigated the role the shell structure in *Adansonia* ecology may play. Both, the African *A. digitata* and the Australian *A. gregorii* are species of fire-prone habitats possessing a well-adapted bark to withstand wildfires (Owen 1974; Bowman 1997; Sidibé and Williams 2002; Chapotin et al. 2006; Watson 2007; Nano and Clarke 2011). So far, hydrochory and zoochory gave us ambiguous explanation for the fruit shell’s function.

Our data strongly indicate that the fruit shell of investigated *Adansonia* species serves as adequate protection for the seed from the heat generated in typical savannah wildfires. Even though heat treatment had a positive effect on seed germination compared to no treatment, acid treatment of seeds simulating the passage through digestive tracts of animals resulted in the highest germination rate. The structure of the fruit (size, volume, shell) was found in this study to protect the seed from high temperatures and the resultant increase of temperature was found to subsequently increase germination rates in *A. digitata*. Low density of shell and fruit pulp contributes to good heat insulation for the seeds. High lignin content in the outer exocarp inhibits most likely early ignition of the shell. Our investigation did not reveal the best heat-resistant material in nature; there are e.g., some *Banksia* species with fruits that can survive at least 500 °C for 2 min (Enright and Lamont 1989). Our results show that *A. digitata* and *A. gregorii* are well adapted to fire-prone savannahs.
